# Trimethylamine N-Oxide Generated by the Gut Microbiota Is Associated with Vascular Inflammation: New Insights into Atherosclerosis

**DOI:** 10.1155/2020/4634172

**Published:** 2020-02-17

**Authors:** Yarong Liu, Min Dai

**Affiliations:** ^1^School of Pharmacy, Anhui University of Chinese Medicine, Hefei, Anhui, China; ^2^Key Laboratory of Xin'an Medicine, Ministry of Education, Hefei, Anhui, China; ^3^Anhui Key Laboratory for Research and Development of Traditional Chinese Medicine, Hefei, Anhui, China

## Abstract

Trimethylamine N-oxide (TMAO) is a biologically active molecule generated by the gut microbiota. Accumulating evidences have indicated a close association between high plasma TMAO levels and the risk of developing atherosclerosis (AS). AS is considered a chronic inflammatory disease initiated by vascular endothelial inflammatory injury. Both observational and experimental studies suggest that TMAO can cause endothelial inflammatory injury. However, a clear mechanistic link between TMAO and vascular inflammation of AS is not yet summarized. In this review, we discuss the association between TMAO and AS and focus on the potential role of TMAO in endothelial inflammatory injury. Finally, the utility of TMAO-targeted therapeutic strategies for the treatment of AS is also analyzed.

## 1. Introduction

Within the last decade, cardiovascular disease (CVD) emerged as the leading cause of death worldwide, encompassing multiple disorders such as atherosclerosis (AS) [1]. AS is featured as a chronic inflammatory disease of vascular stenosis due to vascular inflammatory reaction and lipid peroxidation [2, 3]. Indeed, vascular endothelial inflammatory injury is widely considered the initial stage of AS [4].

Furthermore, there is a growing awareness that the gut microbiota is involved in the occurrence and development of AS [5]. The gut microbiota is the collection of microorganisms that inhabit the gastrointestinal tract producing a diverse ecosystem [6]. The homeostasis of the gut microbiota is critical for maintaining human health [7, 8], while gut dysbiosis contributes to the development of various diseases including AS (Table 1) [5]. Using metagenomic analysis of intestinal flora composition, scientists found that the ratio of *Firmicutes* to *Bacteroidetes* in patients with AS is much higher than that in controls [9]. *Actinobacteria* including the genus *Collinsella* is found in atherosclerotic plaques. *Proteobacteria* including the genera *Chryseomonas* and *Helicobacter* is higher in AS patients compared to healthy adults [10]. Besides, the lowered abundance of *Bifidobacteria* and *Lactobacillus* will promote the development of AS [11]. Additionally, *Akkermansia muciniphila* has been proven to be beneficial in the pathogenesis of AS [12]. Scientists profiled a cross-disease cohort of 1250 individuals to observe the gut microbiome and finally found several disease-relevant bacterial species [13]. The authors suggested that the restoration of the healthy gut microbiome might reduce the risks of CVD as well as other related diseases. Besides, the altered microbiome is closely related to inflammatory status of these diseases.

The gut microbiota can transform dietary nutriment to molecules, among which trimethylamine N-oxide (TMAO) has gained much attention due to its potential role as a promoter of AS [14]. TMAO is derived from trimethylamine (TMA). TMA is metabolized by the gut microbiota from choline and L-carnitine; then, it can be transferred into the liver and be oxidized by flavin-containing monooxygenase 3 (FMO3) [15, 16]. The transformation of choline and carnitine to TMAO depends on the balance and diversity of the gut microbiota. Gut dysbiosis can directly lead to high plasma TMAO levels and may result in AS development ultimately [17]. Currently, there are convincing evidences suggesting a direct link between TMAO and the pathogenesis of AS. Moreover, the mechanisms of high TMAO triggering AS have attracted much attention [18–20].

In this review, we have extensively retrospected the publications on the topics of gut microbe-dependent TMAO and AS which were mainly published within the past ten years through PubMed. We have summarized the current state of knowledge about TMAO and discussed the potential causal relationships between TMAO and the development of AS, especially focusing on the impact of TMAO on endothelial inflammatory injury. Finally, we have analyzed the utility of TMAO-targeted therapeutic strategies for the treatment of AS.

## 2. Biosynthesis and Metabolism of TMAO

### 2.1. Chemical Properties of TMAO

**Figure 1:**
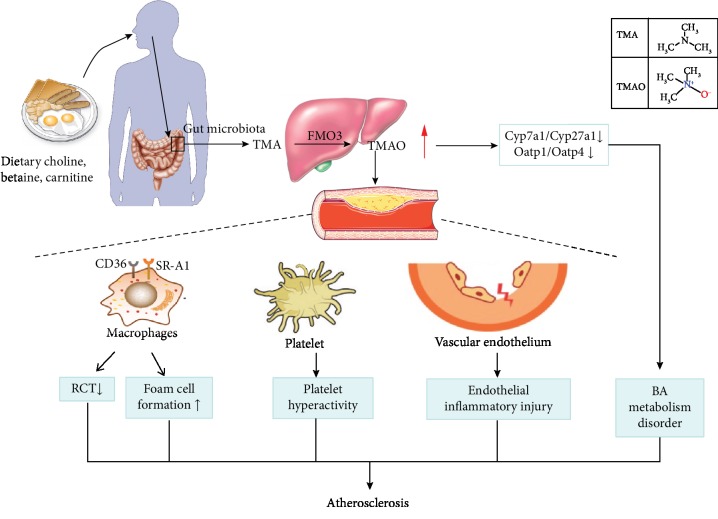
Effects of gut microbiota-dependent trimethylamine N-oxide (TMAO) production on atherosclerosis (AS). The gut microbiota metabolizes dietary choline, L-carnitine, and betaine to form TMA (trimethylamine) and TMAO. TMAO is associated with AS by means of disturbance of bile acid metabolism, inhibition of the RCT, inducement of foam cell formation, activation of platelets, and vascular inflammation. FMO3: flavin-containing monooxygenase 3; Cyp: cytochrome P450; Oatp: organic anion-transporting polypeptide; RCT: reverse transport of cholesterol; BA: bile acids.

TMAO is a quaternary amine compound with the formula (CH_3_)_3_NO and the molecular weight of 75.1 Da. It is a colorless, odorless solid soluble in water and is usually in the form of a dihydrate. Chemically, it is obtained from TMA by oxidation reaction. The structural formulas of TMA and TMAO are shown in Figure 1. TMAO has a lot of important physiological and biochemical functions in the stability of protein structure, as well as osmotic regulation, resistance to ionic instability, and resistance to water pressure [21].

### 2.2. Biosynthesis of TMAO

The biosynthesis of TMAO is influenced by the following three factors. The first one is the composition of food precursors. Evidences exist that the mostly potential sources of TMAO are choline and L-carnitine from daily diet [15, 16]. Dietary sources including red meat, fish, poultry, and eggs are rich in choline [22]. Researches show that higher dietary phosphatidylcholine leads to enhanced plasma TMAO concentrations in mice and humans [5, 23].

The second factor that affects the biosynthesis of TMAO is intestinal microbial activity. TMAO is a cometabolite of the gut microbiota and the host. Dietary choline or L-carnitine is metabolized by the gut microbiota into TMA in the intestine [24]. Research data show that germ-free mice cannot produce TMA and antibiotic treatment of standard mice can decrease TMA formation. Moreover, transplantation of choline-converting bacteria to gnotobiotic mice can increase TMA production and lower serum choline concentration [5, 25, 26]. These studies have confirmed the irreplaceable role of the gut microbiota in TMAO biosynthesis. In addition, eight distinct bacterial strains participating in TMA formation have been identified, including *Anaerococcus hydrogenalis*, *Clostridium asparagiforme*, *Clostridium hathewayi*, *Clostridium sporogenes*, *Edwardsiella tarda*, *Escherichia fergusonii*, *Proteus penneri*, and *Providencia rettgeri* [27] (Table 1).

The third factor that influences TMAO formation is the oxidation of TMA. TMA is a precursor of TMAO, which is transported to the liver and oxidized by flavin-containing monooxygenases (FMO) (Figure 1). There are three members in the FMO family capable of oxidizing TMA to TMAO, in which FMO3 exhibits the highest specific activity [28]. FMO3 hepatic knockdown mice have lower circulating TMAO levels compared to the normal group, suggesting that FMO3 plays an important role in TMAO formation [29]. In addition, researchers show that hepatic FMO3 expression and TMAO circulating levels are lower in male mice compared to female mice. The reason for the gender difference is that androgens inhibit the FMO3 expression. However, the differences are much more modest in humans, because of the varied diet consumed by humans [28]. Besides, FMO3 is modulated by the farnesoid X receptor (FXR), a nuclear receptor activated by bile acid [30].

### 2.3. Metabolism of TMAO

After the biosynthesis in the liver, TMAO can be distributed homogeneously throughout the body [15, 23, 31]. TMAO is a small molecule and is readily filtered by the kidneys [32]. A part of TMAO is reduced to TMA by bacterial TMAO reductase in the human gut [33]. TMA and TMAO are excreted mainly in urine within 24 h, as well as in sweat and exhaled air [20]. Research shows that over 95% of TMAO is eliminated by the kidney in the prototype form [34].

## 3. TMAO in Atherosclerosis Development

There are accumulating evidences suggesting an association between TMAO and the risk of developing AS [5, 35]. Below, we discuss this association from the perspectives of human trials, animal experiments, and cell culture.

### 3.1. Human Trials Verifying the Correlation between TMAO and AS

People at risk of AS have higher plasma levels of TMAO compared to healthy people, as well as higher concentrations of the TMAO precursors in plasma, such as choline and L-carnitine (Table 2).

Higher dietary betaine intake was associated with a nonlinear higher risk of incident coronary heart disease in 3,924 African Americans [36]. A follow-up study included 229 CVD patients and 751 randomly selected healthy participants in a Mediterranean population. The results of this study indicated that plasma concentrations of five metabolites in the choline pathway were associated with increased risks of major adverse cardiovascular events [37]. In patients (*n* = 2,595) undergoing cardiac evaluation, choline and L-carnitine in plasma predicted increased risks of major adverse cardiac events [25]. In the clinical outcome study of 4,007 participants, increased plasma concentrations of TMAO were associated with enhanced risks of major adverse cardiovascular events during the 3-year follow-up [23]. In a cross-sectional study of 227 patients, higher serum TMAO levels may be associated with an increased number of infarcted coronary arteries in patients who undergo cardiovascular surgery [38]. Additionally, in a 5-year follow-up study, patients with stable coronary artery disease participated in the experiment. The results showed that elevated plasma TMAO levels were associated with incident mortality and artery infarction [39]. Another report indicated that urinary TMAO was correlated with the risks of coronary heart diseases (*n* = 275) [40].

However, there are still some opposite results about the correlation between TMAO and AS. The plasma levels of TMAO in humans correlate positively with an increase in age. In a cohort of subjects (*n* = 817) of ages 35-55 over a 10-year follow-up, there was no significant association between TMAO concentrations and AS progression [41]. This study was conducted in relatively younger and healthier individuals as compared to other studies. Based on this result, we speculate that because these early-middle-age patients were mostly in the early stages of AS progression, the cardiovascular effects of TMAO have not yet emerged. Besides, TMAO is excreted in urine. Kidney function in younger individuals is better than that in older ones; therefore, TMAO can be cleared out of the body in time. These results indicate that the conclusion of high TMAO correlating with AS is persuasive after excluding age-related factors. Besides, it is reported that TMAO levels in plasma are confounded by impaired kidney function and poor metabolic control but are not associated with the presence of coronary heart disease [42]. In addition, researches performed a case-control study of patients with large-artery atherosclerotic ischemic stroke and TIA (transient ischemic attack) [43]. There was no obvious change of blood TMAO levels in asymptomatic AS, which is in the early stages of AS progression. Besides, the TMAO levels decreased in stroke and TIA patients compared to controls. As the authors postulated, it was the stroke event and the treatment that reduced TMAO levels. Another report also indicated no remarkable association between TMAO and carotid AS [44]. There were some limitations in this analysis including the use of nonfasting samples, lack of information of vegetarians versus omnivores, incomplete consideration about patient inclusion, and the small event rate.

To summarize this part, differences in the study designs, clinical contexts, geographic and ethnic backgrounds, or dietary habits may alter the role of TMAO in AS progression. Therefore, in the further human trials, demographics, genetic factors, medication, disease staging, dietary habits, and food intake of the participants should be taken into consideration.

### 3.2. Animal Experiments Revealing the Causal Relationship between TMAO and AS

Mice treated with TMAO precursors or TMAO develop more AS (Table 3). Research data showed that heart failure severity was enhanced significantly in mice fed with additional choline or TMAO before surgical transverse aortic constriction [45]. The AS plaque area was increased in ApoE^−/−^ mice fed with additional choline or TMAO compared to mice fed with a control diet, while antibiotic treatment could reverse this trend [5]. Those results suggested a causal relationship between TMAO and AS and an irreplaceable role of the gut microbiota in the metabolism of choline. Furthermore, TMAO directly increased the platelet hyperreactivity *in vivo*. In FeCl_3_-induced carotid artery injury mice, thrombus formation was increased along with plasma TMAO concentrations compared to the control group. The platelet aggregation was significantly increased when mice were fed a chemically defined diet supplemented with either 0.12% TMAO or 1% choline [26]. A study proposed that interleukin 23 (IL-23) and its downstream target IL-22 relieved AS by inhibiting TMAO in mice [46].

Similar to human trials, studies in mice provided evidences that age factor must be taken into account in the research of CVD. Recent study has verified that plasma TMAO levels were higher in mice at 20-24 months of age than those at 8-10 weeks of age. Besides, endothelial dysfunction and oxidative stress are elevated with aging [47].

However, there are still some studies giving the opposite results. A recent study showed no association between TMAO and the risk of AS [48]. In this study, the researchers supplemented choline to ApoE^−/−^ mice at 8 weeks of age instead of 4 weeks. We consider that this conflicting result may be due to timing of choline dietary interventions. The atherosclerotic disease already starts to develop at 8 weeks of age; thus, the change of TMAO may have no obvious effect on the development of AS. In addition, another report suggested that TMAO slowed aortic lesion formation in ApoE^−/−^ mice [49]. In this article, we note that male ApoE^−/−^ mice were transfected with an adeno-associated viral vector containing the human CETP gene (cholesteryl ester transfer protein). This gene plays an important role in reverse cholesterol transport and may reduce cardiovascular damage caused by TMAO. The third study giving the opposite results is performed in LDLR^−/−^ mice [50]. This study showed that dietary choline, betaine, or TMAO supplementation did not induce AS development, despite increasing the plasma TMAO concentrations. The reasons for the discrepancy may be due to the mouse model and housing conditions.

### 3.3. Cell Culture-Based Studies Illuminating the Mechanisms of TMAO-Induced AS

Several studies in cell culture provide key evidences about the mechanisms of TMAO inducing AS (Table 3). As demonstrated in Figure 1, TMAO promotes the occurrence and development of AS mainly through four approaches, including disturbance of bile acid (BA) metabolism, inhibition of the reverse transport of cholesterol (RCT), activation of platelets, and vascular inflammation.

A study conveyed in peritoneal elicited macrophages and RAW264.7 showed that TMAO increased the expression of scavenger receptors on macrophages and caused alternations in BA metabolism in the liver and intestine [51]. Researchers exposed mouse peritoneal macrophages to TMAO *in vitro* and found that TMAO suppressed the RCT and decreased levels of liver BA synthesis, as well as the levels of BA transporters [25]. Another study indicated that TMAO could cause AS via excess cholesterol accumulation and foam cell formation by means of increasing the mRNA levels of cluster of differentiation 36 (CD36) and scavenger receptor A1 (SR-A1) [5]. Zhu et al. [26] verified that direct exposure of platelets to TMAO could enhance platelet activation by increasing the release of Ca^2+^ from intracellular stores. In addition, data showed that TMAO had an impact on endothelial inflammatory injury [52], which will be discussed in the following paragraphs detailedly.

Above all, a close association between TMAO and the risk of developing AS is verified in both observational and experimental studies. Besides, the mechanisms of TMAO promoting AS are illustrated by several studies as above. However, we should still be cautious to conclude that TMAO causes AS, because there are important differences in atherogenesis between mice and humans [53]. For humans, most of the plasma cholesterol is contained in low-density lipoprotein (LDL) particles, whereas most of their cholesterol is in high-density lipoprotein (HDL) particles for mice. Compared to humans, mice can hardly transfer cholesterol ester from HDL to very low-density lipoprotein (VLDL) and LDL [32]. Therefore, more evidences need to be provided to further illuminate the causal relationship between TMAO and AS in human trials.

## 4. TMAO Induces Endothelial Inflammatory Injury

AS is considered a chronic inflammatory disease initiated by vascular endothelial inflammatory injury [2, 3]. In recent years, TMAO is identified as a predictor of AS using a metabolomics approach in the clinical studies as we discussed above. Moreover, TMAO is shown to have an impact on endothelial inflammatory injury in both *in vivo* studies and *in vitro* studies [31, 54] (Table 4).

### 4.1. TMAO Promotes the Release of Inflammatory Cytokines

TMAO treatment induces the release of inflammatory cytokines, contributing to the development of AS. Researches verified that the process of aging increased circulating TMAO levels, which may increase the expression of tumor necrosis factor-*α* (TNF-*α*), IL-1*β*, and IL-10 [18, 55]. In human umbilical vein endothelial cells (HUVECs), TMAO enhanced the levels of IL-1*β* and IL-18 in a dose-and time-dependent manner [56]. In carotid artery endothelial cells (CAECs), TMAO activated the nod-like receptor family pyrin domain containing 3 (NLRP3) inflammasomes, thereby inducing endothelial hyperpermeability and promoting the release of inflammatory cytokines [57]. Additionally, in LDLR^−/−^ mice, acute TMAO injection led to the upregulated inflammatory markers and the activated inflammatory pathways, including MAPK and NF-*κ*B signaling pathways. These observations were recapitulated in human aortic endothelial cells (HAECs) and vascular smooth muscle cells (VSMCs) [54].

### 4.2. TMAO Enhances Inflammation Adhesion

Results demonstrated that TMAO induced the pathological process of AS by accelerating endothelial dysfunction, including increasing monocyte adhesion. Studies verified that TMAO promoted the recruitment of activated leukocytes to endothelial cells via the activation of MAPK and NF-*κ*B signaling pathways [54]. In addition, TMAO could upregulate the expression of vascular cell adhesion molecule-1 (VCAM-1) though the activation of the PKC/NF-*κ*B pathway, which ultimately increased monocyte adhesion [52].

### 4.3. TMAO Promotes Oxidative Stress

Several studies showed that high plasma TMAO could cause endothelial dysfunction and vascular inflammation due to promoting oxidative stress [55, 56, 58, 59]. TMAO significantly triggered oxidative stress by inhibiting the activity of endothelial nitric oxide synthase (eNOS) and production of nitric oxide (NO) via the activation of the TXNIP-NLRP3 inflammasome [56]. Studies about age-related CVD also verified that TMAO triggered oxidative stress. Circulating TMAO levels were elevated with age, which may increase vascular inflammation and oxidative stress by impairing eNOS-derived NO bioavailability [55]. Elevated circulating TMAO during the aging process may increase oxidative stress because of repression of SIRT1 expression and activation of the p53/p21/Rb pathway [59].

### 4.4. The Mechanisms of TMAO Promoting Endothelial Inflammatory Injury

**Figure 2:**
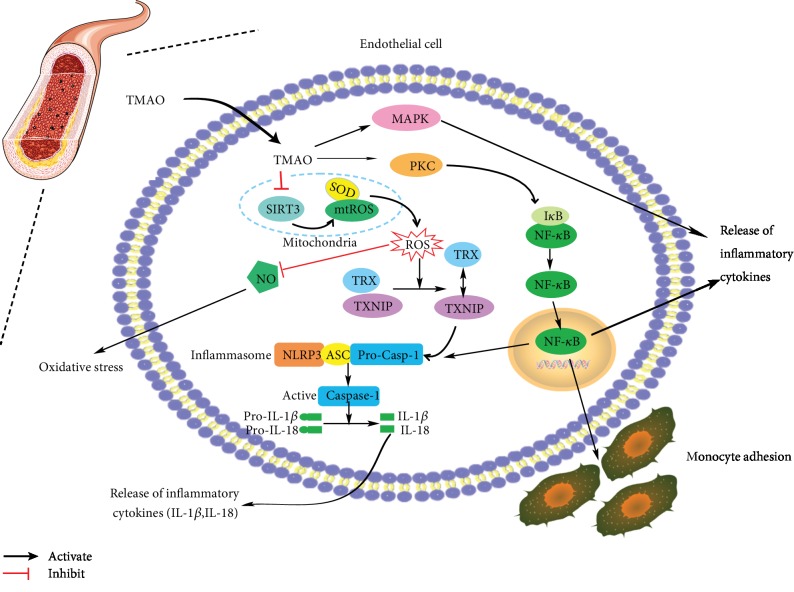
The mechanisms of TMAO (trimethylamine N-oxide) inducing endothelial inflammatory injury. TMAO can promote the release of inflammatory cytokines, enhance the monocyte adhesion to the endothelial cells, and promote the oxidative stress. IL-1*β*: interleukin 1*β*; IL-18: interleukin 18; NLRP3: the nod-like receptor family pyrin domain containing 3; SIRT3: sirtuin 3; SOD2: superoxide dismutase 2; mtROS: mitochondrial reactive oxygen species; ROS: reactive oxygen species; PKC: protein kinase C; NF-*κ*B: nuclear factor-*κ*B; NO: nitric oxide; TRX: thioredoxin; ASC: apoptosis-associated speck-like protein; caspase-1: cysteinyl aspartate-specific proteinase-1; TXNIP: thioredoxin-interacting protein; MAPK: mitogen-activated protein kinase.

In conclusion of the studies above, we have summarized the mechanisms of TMAO promoting endothelial inflammatory injury as follows (Figure 2).

The first approach is TMAO-mediated mtROS production and activation of the NLRP3 inflammasomes. After the biosynthesis in the liver, TMAO goes into the blood vessels and inhibits sirtuin 3 (SIRT3). Sirtuins are NAD^+^-dependent enzymes. SIRT3 directly binds to and deacetylates SOD2. Thereby, inhibition of SIRT3 decreases SOD2 activity and then leads to a significantly bad effect on mtROS homeostasis [60, 61]. Subsequently, elevated ROS production leads to inhibition of NO release, thus contributing to oxidative stress [55]. Besides, elevated ROS production induces the dissociation of thioredoxin-interactive protein (TXNIP) from thioredoxin (TRX). As a consequence, TXNIP activates the NLRP3 inflammasome, thus upregulating IL-1*β* and IL-18 [56, 62].

The second approach is activation of the NF-*κ*B signaling pathway by TMAO. TMAO activates protein kinase C (PKC) and consequently contributes to the activation of the NF-*κ*B pathway [52]. After the activation of PKC, I*κ*B is degraded by phosphorylation and the trimer is disintegrated to release the activated NF-*κ*B [63]. Due to the activation of the NF-*κ*B pathway, a cascade of cellular inflammatory responses occurs, including the release of inflammatory cytokines and monocyte adhesion with the vascular endothelium.

And last but not least, TMAO inhibits the activity of MAPK/ERK, reduces the activation of the NF-*κ*B pathway, and consequently decreases the inflammatory response [54, 64].

However, there are some studies providing an opposite result. In a cohort of 31 patients who underwent hemodialysis, high plasma TMAO levels induced by oral L-carnitine supplementation could decrease markers of vascular injury and oxidative stress such as ICAM-1, VCAM-1, and malondialdehyde (MDA) levels [65]. Some limitations in this article were responsible for these results. Age and gender were not taken into account in this article. This study did not change the participants' lifestyles; thus, it could not totally exclude the medication interference. Besides, this study has small sample size with high dropout rates. Another study providing an opposite result was conducted among 271 healthy adults. This study suggested that TMAO concentrations were not related to C-reactive protein (CRP) or IL-6 concentrations, but with TNF-*α*, and two soluble TNF receptors [19]. The reason for the opposite result may be that the long-term dietary habits differed among the participants and the food intake was hard to assess. The third opposite results indicated no relation between TMAO and any oxidative stress markers [66]. This conclusion was based on 20 healthy aged women. The authors should increase the number of participants for eliminating distractions.

To better explore the mechanisms that TMAO develops AS and induces endothelial inflammatory injury, researchers should pay more attention to details about experimental setups, such as strains, genders, diet, and housing conditions of laboratory animals. However, in consideration of the difference between the mouse model and humans, more researches and data of human trials should be provided to support these mechanisms. Besides, human trials need to follow the recommendations we made in the previous section.

## 5. Therapeutic Strategies

According to the discussion above, TMAO is a metabolite produced by the gut microbiota and is one of the causes of AS. This link between TMAO and AS provides an opportunity to identify new therapeutic targets to ameliorate vascular inflammation and cure the diseases. Several therapeutic strategies of decreasing TMAO levels are summarized in Table 5 [67].

### 5.1. Therapies Targeting the Gut Microbiota to Reduce TMAO Generation

The use of prebiotics and probiotics could be helpful to elicit a positive impact on gut microbiota composition, resulting in the regulation of TMAO generation [68]. Prebiotics are nondigestible food components that have beneficial effects on the host by selectively stimulating the growth and activity of beneficial bacteria, while probiotics are living microbes capable of producing beneficial effects on human health. Mice colonized with human infant flora had a reduced TMA production [69]. Other studies have initiated the use of archaea to deplete TMA and TMAO, because these archaea use methyl compounds such as TMA and TMAO as the substrate to generate methane [70, 71]. *Lactobacillus* and *Bifidobacterium* genera are used as probiotics to reduce the extent of AS and bring comprehensive benefits to life [72]. Recently, it is reported that probiotics significantly promoted the anti-inflammatory cytokine IL-10 and decreased proinflammatory cytokines such as IL-1*β* [73]. The mechanisms that probiotics prevent AS can be concluded as modulation of the gut microbiome and regulation of miRNA [74]. Considering the gut microbiota which is responsible for the synthesis of TMAO, we can find and utilize some probiotic strains to prevent AS. The weakness of this strategy is that the treatment effects vary from person to person, because gut microbiota composition is affected by a variety of factors.

The use of antibiotics to eliminate the microbiota has also been devised in treating CVD. Antibiotics can prevent dietary precursors (choline, betaine, and L-carnitine) being transformed into TMA. The use of oral broad-spectrum antibiotics such as ciprofloxacin and metronidazole suppressed TMAO levels in a human trial [23]. However, the levels of TMAO were detectable one month after taking the antibiotics. A mix use of vancomycin, neomycin-sulphate, metronidazole, and ampicillin in mice unveiled an inhibition of plasma TMAO levels and inhibition of macrophage foam cell formation [5]. However, the chronic application of antibiotics is not viable since it can lead to bacterial resistance and inhibition of the beneficial bacteria. Thus, additional researches are needed to ensure the safety of antibiotics.

### 5.2. Therapies Targeting TMA to Reduce TMAO Generation

Another appealing approach is the inhibition of TMA biosynthesis. 3,3-Dimethyl-1-butanol (DMB), an analogue of choline that inhibits choline TMA lyase, can reduce plasma TMAO levels and attenuate the development of choline diet-enhanced AS [18, 75]. However, DMB is not able to avoid the TMAO synthesis completely. Another report proposed the use of meldonium, a compound used as an anti-ischemic and antiatherosclerotic drug [76]. It seemed to reduce plasma TMA levels in humans by increasing its urinary excretion and reducing its biosynthesis from L-carnitine [77]. However, meldonium reduces TMA formation from L-carnitine, but not from choline. Another study examined the TMA-forming-related gut microbiota and found that choline TMA lyase (cutC) and carnitine oxygenase (cntA) amplicons were related to various taxa [78]. However, these experimental results need further investigation before clinical application. In addition, plant sterol esters (PSEs) were also identified to attenuate cholesterol accumulation and prevent AS by inhibiting microbial production of TMA in ApoE^−/−^ mice [79].

There is another option, FMO3 enzyme inhibitor, which is able to cut down the transformation of TMA into TMAO. Hepatic knockdown of FMO3 in mice using an antisense oligonucleotide decreased circulating TMAO levels and attenuated AS [30, 80]. But this treatment is hard to replicate in humans, because FMO3 is involved in the oxidative metabolism of a large number of drugs, exogenous substances, and other chemicals in the body, not just TMAO. Another problem would be that after hepatic knockdown of FMO3, TMA will be accumulated in plasma resulting in trimethylaminuria and causing some new diseases.

### 5.3. Herbal Products Reducing TMAO Generation

Several herbal products reveal an impact on decreasing plasma TMAO levels. *Gynostemma pentaphyllum*, a plant used as a traditional Chinese medicine to treat hyperlipidemia and obesity, seemed to reduce plasma TMAO levels in rats [81]. Besides, Gancao, the root of *Glycyrrhiza uralensis*, seemed to reduce TMAO levels when Fuzi (the processed lateral root of *Aconitum carmichaelii*) was coadministered [82]. Some researches proposed resveratrol, a natural polyphenolic compound in the diet, which could modulate gut composition, decreasing TMA-forming bacteria and increasing the beneficial bacteria [83]. Oolong tea extract and citrus peel polymethoxyflavones could reduce TMAO production in L-carnitine-feeding mice [84]. Berberine (BBR) is used for antigastrointestinal tract infection clinically. In male ApoE^−/−^ mice fed with high-fat diet, BBR could change the abundances of *Firmicutes* and *Verrucomicrobia* and reduce the expression of hepatic FMO3 and serum TMAO levels markedly [85]. Another natural pharmaceutical ingredient, *trigonelline*, a compound from *Trigonella foenum-graecum*, inhibited the formation of TMAO from TMA by inhibiting FMO3 [86]. However, the effects of herbal products are mostly verified in mice, not in humans.

In conclusion, the therapeutic strategies mentioned above have certain limitations or shortcomings. In the future, new therapeutic targets based on the role of TMAO in vascular inflammation could lead to a promising therapeutic avenue in the treatment of AS. Herbal products are a treasure with multicomponent and multitarget, from which we can dig out new therapeutic strategies reducing TMAO generation.

## 6. Conclusion and Perspective

AS is featured as a chronic inflammatory disease with vascular stenosis due to lipid peroxidation and endothelial inflammatory injury [2, 3]. In recent years, increasing evidences have suggested an important role of the gut microbiota and its metabolite, TMAO, in the development of AS. The findings have shed light on the great potential of targeting TMAO to elucidate the fundamental mechanisms underlying the disease and to provide a new therapeutic strategy. However, it is worth noting that excessive inhibition of TMAO may also have some adverse effects [87]. In the future, we need to establish adequate TMAO-targeting therapeutic strategies for AS.

Currently, most of the present data from clinical and epidemiological researches do not explain the mechanisms that TMAO causes AS, whereas studies in most animal models and cell culture have provided key evidences supporting the mechanisms. However, due to the important differences in atherogenesis between mice and humans, we still need to elucidate these mechanisms further in human experiments. Furthermore, in the further human trials, more details on experimental designs should be taken into consideration, such as demographics, genetic factors, medication, disease staging, dietary habits, and food intake of the participants. In a word, there is a long way to go before the unequivocal establishment of TMAO-targeted therapies for AS in the clinic.

## Figures and Tables

**Table 1:** The most important microbiota that influences AS and TMA production [9–13, 27, 88].

Phyla	Genus	Species	AS	TMA production
*Actinobacteria*	*Collinsella*		√	
	*Alistipes*	*Shahii*	√	
	*Bifidobacterium*	*Bifidobacteria*	√	

*Bacteroidetes*	*Bacteroides*		√	
	*Prevotella*		√	
	*Porphyromonas*	*Gingivalis*	√	

*Chlamydiae*	*Chlamydia*	*Pneumoniae*	√	

*Firmicutes*	*Anaerococcus*	*Hydrogenalis*	√	+
	*Anaeroglobus*		√	
	*Eubacterium*		√	
	*Clostridium*	*Asparagiforme*	√	+
	*Clostridium*	*Hathewayi*	√	+
	*Clostridium*	*Sporogenes*	√	+
	*Lactobacillus*	*Salivarius*	√	
	*Solobacterium*	*Moorei*	√	
	*Faecalibacterium*	*cf. prausnitzii*	√	
	*Atopobium*	*Parvulum*	√	
	*Roseburia*		√	
	*Ruminococcus*	*Gnavus*	√	
	*Streptococcus*	spp.	√	

*Proteobacteria*	*Chryseomonas*		√	
	*Escherichia*	*Coli*	√	
	*Klebsiella*	spp.	√	
	*Enterobacter*	*Aerogenes*	√	
	*Eggerthella*	*Lenta*	√	
	*Helicobacter*		√	
	*Neisseria*		√	
	*Salmonella*		√	
	*Shigella*		√	
	*Edwardsiella*	*Tarda*	√	+
	*Escherichia*	*Fergusonii*	√	+
	*Proteus*	*Penneri*	√	+
	*Providencia*	*Rettgeri*	√	+

*Verrucomicrobia*	*Akkermansia*	*Muciniphila*	√	

AS: atherosclerosis; TMA: trimethylamine; √: microbiota that influences the occurrence and development of AS; +: microbiota that participates in the TMA formation in the presence of choline.

**Table 2:** Human trials studying the association between TMAO and AS.

	Subject	Population	Age (years)	Gender	Indicator	Main findings	Duration (years)	References
Positive results	Healthy African American participants	3,924	37-59	Male (33.4%)	CIMT; CAC; abdominal aortic calcium and left ventricular mass	(1) In women, higher choline intake was associated with lower left ventricular mass and abdominal aortic calcium score (2) Betaine intake was associated with greater risk of incident CHD	9	[36]
	CVD patients and healthy adults	Cases 229 and noncases 751	55-80	Male (41.9%)	5 metabolites in the choline pathway	Plasma metabolites from the choline pathway were associated with an increased risk of CVD in a Mediterranean population	4.8	[37]
	Patients undergoing cardiac evaluation	2,595	54-71	Male (70%)	Levels of L-carnitine and TMAO in plasma and urine	(1) There was a dose-dependent association between carnitine concentration and risk of CVD (2) Carnitine concentration predicted an incident (3-year) risk of major adverse cardiac events (3) TMAO was the primary driver of the association of carnitine with CVD risk	3	[25]
	Patients undergoing elective coronary angiography	4,007	52-74	Male (64%)	Levels of TMAO in plasma and urine; plasma choline and betaine	Increased plasma concentrations of TMAO were associated with increased risks of major adverse cardiovascular events	3	[23]
	Patients undergoing cardiovascular surgery	227	61–74	Male (70%)	Levels of TMAO in serum; number of infarcted coronary arteries	Higher serum TMAO levels were associated with an increased number of infarcted coronary arteries	—	[38]
	Patients with stable coronary artery disease	2,235	52-74	Male (71%)	Levels of TMAO in plasma	Elevated concentrations of plasma TMAO were associated with incident mortality and artery infarction, independent of other traditional risk factors in stable cardiac patients	5	[39]
	Urban Chinese adults in a nested case-control study	275 patients with CHD and 275 controls	62.2 ± 8.7	Male (46%)	Urinary levels of TMAO	Urinary TMAO was correlated with the risk of CHD	10	[40]
	Multiethnic participants	271	42-63	Male (64.9%)	Levels of TMAO in serum	An association of TMAO with prevalent CVD in a multiethnic population	—	[89]

Negative results	Healthy participants	817	33-55	Male (52%)	Levels of TMAO in plasma; CIMT; CAC	TMAO may not contribute significantly to advancing early AS risk among healthy early-middle-aged adults	10	[41]
	Patients of suspected CAD	339	55-71	Male (68%)	Plasma TMAO or betaine levels	Plasma levels of TMAO were not associated with the history, presence, or incidence of CVD events	8	[42]
	Patients with large-artery atherosclerotic ischemic stroke and TIA	322 patients and 231 controls	56-61	Male (63.2%)	Plasma TMAO	(1) There was no obvious change of blood TMAO levels in asymptomatic AS (2) TMAO levels were decreased in stroke and TIA patients	1	[43]
	Patients with carotid artery AS	264 patients and 62 controls	67.6 ± 8.4	Male (68.6%)	Circulating levels of carnitine-related metabolites	Patients with carotid AS had increased serum levels of carnitine, but not TMAO	10	[44]

AS: atherosclerosis; CVD: cardiovascular disease; TMAO: trimethylamine N-oxide; CAC: coronary artery calcium; CIMT: carotid intima-media thickness; CHD: coronary heart disease; TIA: transient ischemic attack; CAD: coronary artery disease.

**Table 3:** Animal experiments and cell culture studying the causal relationship between TMAO and AS.

	Experimental models	Intervention	Main observations	References
Positive results	C57BL6/J mice	Feeding with choline- or TMAO-enriched diet before transverse aortic constriction	Either TMAO- or choline-enriched diets enhanced heart failure severity	[45]
	ApoE^−/−^ mice Germ-free mice	Feeding with additional choline	The AS plaque area was increased compared with mice fed with a control diet	[5]
	Peritoneal macrophages	The mice were fed a diet with TMAO, betaine, or choline	(1) The mRNA levels of CD36 and SR-A1 were increased (2) The macrophages had excess cholesterol accumulation and foam cell formation	[5]
	ApoE^−/−^ mice	Redundant L-carnitine or antibiotics were introduced into the diet	TMAO could suppress RCT and levels of liver BA synthetase and BA transporters and modulate the activity of cholesterol transporters in macrophages	[25]
	LDLR^−/−^ mice	Chronic choline supplementation	Plasma TMAO concentrations were increased and inflammatory gene expression in vascular cells was increased	[90]
	FeCl_3_-induced carotid artery injury mice Germ-free mice	i.p. TMAO or feeding with diet (0.12% TMAO or 1% choline supplementation)	(1) Intestinal flora promoted the conversion of choline to TMAO (2) There was an association of choline, TMAO, and thrombus risk	[26]
	Platelets	Platelets were exposed to TMAO	TMAO could enhance platelet activation from multiple agonists by increasing the release of Ca^2+^ from intracellular stores	
	LDLR^−/−^ mice ApoE^−/−^ mice Il23^−/−^ mice, Il22^−/−^ mice	The mice were fed with Western diet	IL-23 and its downstream target IL-22 relieved AS by inhibiting TMAO	[46]
	ApoE^−/−^ mice/peritoneal macrophages and RAW264.7	The mice were fed a high-fat diet with or without TMAO for 8 weeks/the cells were treated with TMAO or ox-LDL	(1) TMAO promoted the AS *in vivo* and *in vitro* (2) The CD36/MAPK/JNK pathway may play a crucial role in TMAO-induced formation of foam cells	[51]
	Mice at 20-24 months of age and mice at 8-10 weeks of age	The mice were treated for 3-4 weeks with broad-spectrum poorly absorbed antibiotics	(1) The gut microbiota was an important mediator of age-related arterial dysfunction (2) Plasma TMAO was higher in aged mice (3) Endothelial dysfunction and oxidative stress were elevated with aging	[47]

Negative results	ApoE^−/−^ mice	The mice were supplemented with choline at 8 weeks of age	No association was observed between TMAO and the risk of AS	[48]
	ApoE^−/−^ mice	The mice were transfected with an adeno-associated viral vector containing the human CETP gene	TMAO slowed the aortic lesion formation in ApoE^−/−^ mice	[49]
	LDLR^−/−^ mice ApoE^−/−^ mice	Dietary intervention using extra choline, betaine, or TMAO	Dietary choline, betaine, or TMAO supplementation did not induce AS development	[50]

AS: atherosclerosis; TMAO: trimethylamine N-oxide; ApoE^−/−^: apolipoprotein E-deficient; RCT: reverse cholesterol transport; BA: bile acids; LDLR^−/−^: lipoprotein receptor-deficient; ADP: adenosine diphosphate; RAW264.7: a macrophage cell line; ox-LDL: low-density lipoprotein; CD36: cluster of differentiation 36; IL: interleukin; MAPK: mitogen-activated protein kinases; JNK: c-Jun N-terminal kinase; SR-A1: scavenger receptor A1; CETP: cholesteryl ester transfer protein.

**Table 4:** Researches about TMAO inducing endothelial inflammatory injury.

	Experimental models	Results	Proposed mechanisms	References
Positive results	CAECs	TMAO induced IL-1*β* production	↑ NLRP3 inflammasomes ↑ Endothelial hyperpermeability	[57]
	HUVECs/ApoE^−/−^ mice	TMAO promoted the release of IL-1*β*	↓ SIRT3-SOD2-mitochondrial ROS signaling pathway ↑ NLRP3 inflammasome	[18]
	FHCs	TMAO contributed to colonic epithelial inflammation and promoted the release of inflammatory cytokines	↓ ATG16L1-induced autophagy ↑ NLRP3 inflammasome	[91]
	LDLR^−/−^ mice/HAECs/HVSMCs	TMAO promoted the release of inflammatory cytokines and promoted the recruitment of activated leukocytes to endothelial cells	↑ MAPK and NF-*κ*B signaling pathway	[54]
	THP-1/HUVECs	TMAO decreased endothelial self-repair and increased monocyte adhesion	↑ PKC/NF-*κ*B/VCAM-1 pathway	[52]
	HUVECs	TMAO augmented the release of IL-1*β* and IL-18 and triggered oxidative stress	↑ TXNIP-NLRP3 inflammasome	[56]
	Rats	TMAO increased the release of TNF-*α* and IL-1*β* and promoted oxidative stress	↓ eNOS-derived NO production in the aorta	[55]
	Human/SAMR1 mice/SAMP8 mice/HUVECs	TMAO increased oxidative stress both *in vivo* and *in vitro*	↓ SIRT1 expression ↑ p53/p21/Rb pathway	[59]
	HUVECs	TMAO enhanced the protein expression of p65, p-p65, lamin A, lamin C, ERK, p-ERK, p38, p-p38, and COX-2; TMAO induced the proliferation and apoptosis of HUVECs	↑ NF-*κ*B/MAPK pathways	[64]

Negative results	31 HD patients with carnitine deficiency	Oral L-carnitine supplementation was associated with increased TMAO levels, whereas it decreased ICAM-1, VCAM-1, and MDA levels	Limitations: age, gender, and lifestyles of the participants were not taken into account, especially medication interference	[65]
	271 healthy adults, ≥18 years old	Augmented TMAO levels in plasma led to an overexpression of TNF-*α* and two soluble TNF receptors but were not related to CRP or IL-6	Limitations: the long-term dietary habits differed among the participants and the food intake was hard to assess	[19]
	20 healthy aged women	No relation between TMAO and any oxidative stress markers	Limitations: the number of participants was small	[66]

TMAO: trimethylamine N-oxide; TNF-*α*: tumor necrosis factor-*α*; IL-1*β*: interleukin 1*β*; IL-10: interleukin 10; CAECs: carotid artery endothelial cells; NLRP3: the nod-like receptor family pyrin domain containing 3; HUVECs: human umbilical vein endothelial cells; ApoE^−/−^: apolipoprotein E-deficient; SIRT3: sirtuin 3; SOD2: superoxide dismutase 2; ROS: reactive oxygen species; FHCs: fetal human colon cells; ATG16L1: autophagy-related protein; LDLR^−/−^: lipoprotein receptor-deficient; HAECs: human umbilical artery endothelial cells; HVSMCs: human vascular smooth muscle cells; PKC: protein kinase C; NF-*κ*B: nuclear factor-*κ*B; VCAM-1: vascular cell adhesion molecule-1; eNOS: endothelial nitric oxide synthase; NO: nitric oxide; TXNIP: thioredoxin-interacting protein; SAMR1 mice: senescence-accelerated mouse resistance 1; SAMP8 mice: senescence-accelerated mouse prone 8; HD: hemodialysis; ICAM-1: intercellular cell adhesion molecule-1; MDA: malondialdehyde; ERK: extracellular signal-regulated kinase; COX: cyclooxygenase.

**Table 5:** Therapeutic strategies targeting the reduction of TMAO generation.

	Therapy	Effects	Weakness	References
Targeting the gut microbiota	Prebiotics	Gut microbiota composition was improved, thus decreasing TMAO formation	Gut microbiota composition was affected by a variety of factors	[68]
	Probiotics	(1) The use of *Lactobacillus paracasei* reduced TMA production in mice expressing the human baby microbiota (2) Methanogenic bacteria depleted TMAO (3) *Lactobacillus* and *Bifidobacterium* genera are used as probiotics to reduce the extent of AS	The effects were not clear in humans	[69–71]
	Antibiotics	(1) Mixed antibiotics in mice led to an inhibition of plasma TMAO levels and inhibition of macrophage foam cell formation (2) The use of oral broad-spectrum antibiotics suppressed TMAO levels in a human trial	Bacterial resistance and inhibition of the beneficial bacteria	[5, 23]

Targeting TMA formation	DMB	TMA lyase was inhibited, resulting in TMAO formation decrease	Studies performed in mice and rats, not in humans DMB cannot avoid the complete TMAO synthesis	[18, 75]
	Meldonium	Excretion of TMAO was increased though urine; TMAO biosynthesis from L-carnitine was reduced	TMAO formation from choline cannot be reduced	[76, 77]
	CutC/D inhibitors	CutC and cntA amplicons were related to the TMA-forming-related gut microbiota	The experimental results need further investigation	[78]
	PSEs	PSEs attenuated cholesterol accumulation and prevented AS by inhibiting microbial production of TMA in ApoE^−/−^ mice	Studies have been performed in mice only	[79]
	FMO3 enzyme inhibitor	Transformation of TMAO from TMA was inhibited	TMA is accumulated in plasma and causes some new diseases	[30, 80]

Herbal products	*Gynostemma pentaphyllum*	Plasma TMAO levels were reduced in rats	Studies have been performed in rats only	[81]
	Concomitant use of Gancao and Fuzi	Plasma TMAO levels were reduced in rats	Studies have been performed in rats only	[82]
	Resveratrol	Gut microbiota composition was modulated, thus decreasing TMA-forming bacteria and increasing the beneficial bacteria	Studies have been performed in mice only	[83]
	Oolong tea extract and citrus peel polymethoxyflavones	The gut microbiota was remodeled; then, TMAO formation and vascular inflammation were reduced	Studies have been performed in mice only	[84]
	BBR	BBR could change the abundances of *Firmicutes* and *Verrucomicrobia* and reduce the expression of hepatic FMO3 and serum TMAO levels markedly	Studies have been performed in mice only	[85]
	*Trigonelline*	*Trigonelline* inhibited the formation of TMAO from TMA by inhibiting FMO3	Studies have been performed in mice only	[86]

TMAO: trimethylamine N-oxide; TMA: trimethylamine; DMB: 3,3-dimethyl-1-butanol; cutC: choline TMA lyase; cntA: carnitine oxygenase; PSEs: plant sterol esters; FMO3: flavin-dependent monooxygenase 3; BBR: berberine.
